# Gender-related differences and similarities in eligibility for coronary reperfusion and outcome after out-of-hospital cardiac arrest

**DOI:** 10.1186/2197-425X-3-S1-A193

**Published:** 2015-10-01

**Authors:** W Bougouin, F Dumas, E Marijon, G Geri, B Champigneulle, JD Chiche, O Varenne, C Spaulding, JP Mira, X Jouven, A Cariou

**Affiliations:** Cochin Hospital, Medical Intensive Care Unit, Paris, France; Paris Descartes University, Paris, France; Sudden Death Expertise Center, INSERM U 970, Paris, France; Emergency Department, Cochin Hospital, Paris, France; Cardiology Department, European Georges Pompidou Hospital, Paris, France; Cardiology Department, Cochin Hospital, Paris, France

## Background

Out-of-Hospital Cardiac Arrest (OHCA) remains a major public health concern, with low survival. Percutaneous Coronary Intervention (PCI) may improve survival in OHCA of ischemic cause. Selection of patients suitable for early coronary angiography and PCI remains challenging, and differences across gender remains unknown.

## Objectives

To assess the relationship between gender and coronary reperfusion after OHCA, and the relationship between PCI and outcome according to gender.

## Methods

All patients admitted in our center after OHCA were prospectively included in an electronic registry database, from 2000 to 2013. Characteristics were collected according to the Utstein style. We assessed the association between gender and coronary reperfusion (using multivariate logistic regression and propensity-score analysis), and between PCI and outcome (according to gender).

## Results

1817 patients were included (520 women, 29%). Women were older than men (62.8 vs 59.1 years, P < 0.0001), with less cardiomyopathy. They had less frequently bystander cardiopulmonary resuscitation (84% vs 88%, P < 0.05) and less shockable rhythm (42% vs 61%, P < 0.001). After multivariate logistic regression, male sex was associated with the decision to perform coronary angiography (OR 1.73, 95%CI 1.28-2.34, P < 0.001) and results were consistent even after propensity-score matching (P = 0.02). Among 1157 patients who underwent coronary angiography, rate of PCI did not differ between men and women (OR 1.30, 95%CI 0.90 - 1.88, P = 0.17). Results after matching (211 men, 211 women) were consistent (P = 0.13). PCI was associated with favorable outcome by multivariate logistic regression (OR = 1.45, 95%IC = 1.07 - 1.96, *P* = 0.02) with no interaction between gender and PCI (*P* for interaction = 0.40). Association between PCI and outcome was consistent across genders.

## Conclusions

After OHCA, women are less likely to undergo coronary angiography. However rates of PCI after coronary angiography do not differ across gender, and the association between PCI and favorable outcome is similar across gender.

